# Installation Error Calibration Method for Redundant MEMS-IMU MWD

**DOI:** 10.3390/mi16040391

**Published:** 2025-03-28

**Authors:** Yin Qing, Lu Wang, Yu Zheng

**Affiliations:** School of Engineering and Technology, China University of Geosciences (Beijing), Beijing 100083, China; 3002240048@email.cugb.edu.cn (Y.Q.); 2002220053@email.cugb.edu.cn (Y.Z.)

**Keywords:** IMU-MEMS, error calibration, installation error, redundancy technology, MWD

## Abstract

For Measurement While Drilling (MWD), the redundant Micro-Electro-Mechanical Systems Inertial Measurement Unit (MEMS-IMU) navigation system significantly enhances the reliability and accuracy of drill string attitude measurements. Such an enhancement enables precise control of the wellbore trajectory and enhances the overall quality of drilling operations. But installation errors of the redundant MEMS-IMUs still degrade the accuracy of drill string attitude measurements. It is essential to calibrate these errors to ensure measurement precision. Currently, the commonly used calibration method involves mounting the carrier on a horizontal plane and performing calibration through rotation. However, when the carrier rotates on the horizontal plane, the gravity acceleration component sensed by the horizontal axis of the IMU accelerometer in the carrier is very small, which leads to a low signal-to-noise ratio, so that the measured matrix obtained by the solution is dominated by noise. As a result, the accuracy of the installation is insufficient, and, finally, the effectiveness of the installation error compensation is reduced. In order to solve this problem, this study proposes a 45°-inclined six-position calibration method based on the selected hexagonal prism redundant structure for redundant MEMS-IMUs in MWD. Firstly, the compensation matrices and accelerometer measurement errors were analyzed, and the new calibration method was proposed; the carrier of the IMUs should be installed at an inclined position of 45°. Then, six measuring points were identified for the proposed calibration approach. Finally, simulation and laboratory experiments were conducted to verify the effectiveness of the proposed method. The simulation results showed that the proposed method reduced installation errors by 40.4% compared with conventional methods. The experiments’ results demonstrated reductions of 83% and 68% in absolute measurement errors for the *x* and *y* axes, respectively. As a result, sensor accuracy after compensation improved by over 25% compared with traditional methods. The calibration method proposed by this study effectively improves the accuracy of redundant systems, providing a new approach for the precise measurement of downhole trajectories.

## 1. Introduction

For Measurement While Drilling (MWD), the inertial navigation system (INS) is widely used due to its high autonomy and independence. It does not rely on external input information; instead, continuous navigation data are obtained through calculations based on inertial sensors. However, downhole conditions, such as high temperatures and strong vibrations, combined with limitations in manufacturing processes [1,2], pose significant challenges to downhole attitude measurement. These challenges manifest as low precision, poor stability, and inadequate reliability [3]. Additionally, recent studies on MEMS nonlinear dynamics have further revealed the complexity of micro-scale systems under electrostatic actuation [4,5]. These findings emphasize the inherent nonlinearities and environmental sensitivity of MEMS devices, which are particularly critical in downhole applications in which sensor performance is substantially affected by manufacturing tolerances and operational stresses. To improve the reliability and precision of navigation control systems, hardware redundancy technology has been actively developed. This technology involves integrating additional inertial sensors into Inertial Measurement Units (IMUs), resulting in diverse configurations with varying numbers and arrangements of sensors [6,7,8,9]. Redundant inertial navigation systems exhibit various errors, similar to general inertial navigation systems. However, as the number of redundant sensors increases and their configurations become more diverse, the complexity of isolating the deterministic error parameters of the sensors also increases. Moreover, calibration methods employed across different configuration schemes are not universally applicable. Consequently, research on calibration techniques for redundant IMUs is of paramount importance [10,11]. 

In the process of error calibration, the focus is typically on primary parameters such as bias, scale factor, and installation errors. Among these, installation errors significantly impact sensor measurements and the subsequent attitude determination process. These effects become more pronounced as the number of sensors increases, due to the cumulative nature of such errors. Currently, IMU installation errors are commonly addressed through multi-position static tests conducted on rotary tables. For example, WANG Xiong proposed a least-squares quaternion algorithm to compensate for installation errors in MEMS measurement units during wind tunnel experiments. This method was validated using high-precision triaxial rotary table tests [12]. Zheng Zhichao introduced an eight-position self-calibration method for dual-axis inertial navigation systems, which incorporates two additional tilted positions compared with traditional calibration methods [13]. Q. Chen developed a compensation technique for installation errors in pipeline inspection systems, noting that this calibration approach does not require complex or sophisticated experimental equipment [14]. Umar Qureshi simplified traditional correction methods by using gravity as a reference and demonstrated the effectiveness of this calibration approach across different grades of IMUs [15]. The aforementioned studies all focus on the calibration of installation errors, and it can be observed that current research on correcting such errors typically employs gravity as a reference [16,17]. Typically, these methods require mounting the carrier on a horizontal plane and rotating it in 90° increments to align each of the *x*, *y*, and *z* axes vertically, while keeping the other two axes horizontal. The compensation matrix is then derived by mapping the actual measurements from specific points to their theoretical values. Subsequently, this compensation matrix is used to correct the errors. However, it is important to note that due to installation errors, the actual placement of the carrier during measurement is not perfectly vertical but rather nearly vertical. In this scenario, the well inclination angle *I* approaches 90°, resulting in sinI ≈ 0. As a result, the signals measured along the horizontal axes become weak, and the computed measurement matrix is dominated by noise, except for its diagonal elements. Using such a measurement matrix to solve for the compensation matrix inevitably affects the accuracy of the compensation matrix.

In order to solve this problem, this study proposes a novel calibration method for installation errors. This method is based on a redundant structure comprising six MEMS-IMUs, with six specific measurement points selected during the installation of the carrier at a 45° inclination. By analyzing the relationship between the measured and theoretical values, a compensation matrix is derived to correct the installation errors. This approach achieves a higher compensation accuracy than traditional methods, effectively reducing the impact of installation errors on attitude measurement precision. As a result, it significantly improves the overall measurement accuracy of the redundant system, ensuring precise downhole trajectory measurements.

## 2. Inclined Six-Position Installation Error Calibration Method

### 2.1. Redundant Configuration Scheme for MEMS-IMUs

**Figure 1:**
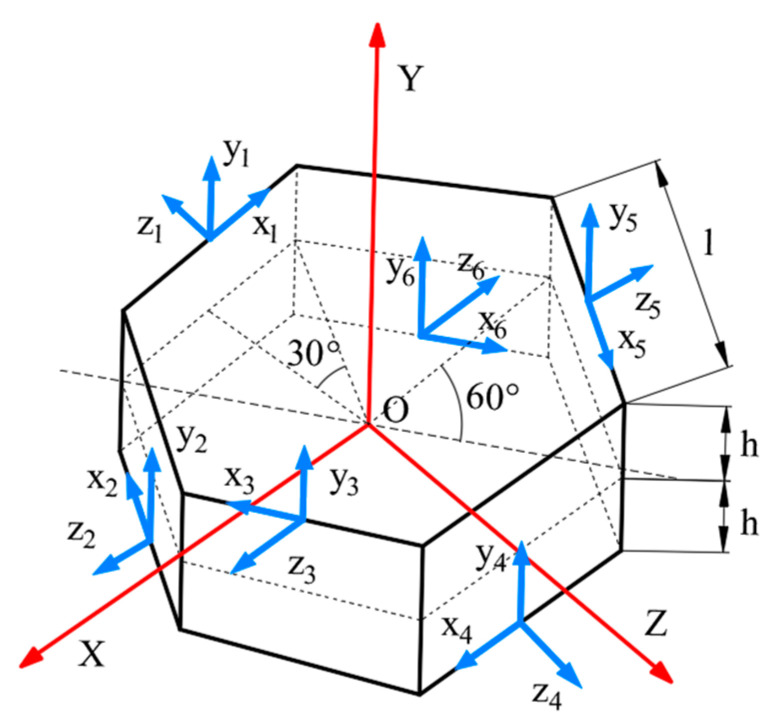
Hexagonal prism MEMS-IMU redundant configuration scheme.

Given that the internal space of downhole drill pipes serves as a circulation channel for drilling fluids, measurement devices used in MWD must feature a hollow structure. To meet this requirement, this study proposes a redundant MEMS sensor configuration scheme. Six MEMS-IMUs are arranged in a regular hexagonal prism structure (as shown in Figure 1), with the IMU-MEMS units mounted on the inner wall of the drill pipe [18].

In a Cartesian coordinate system with the geometric center of the prism as the origin, the *Y* axis is defined as parallel to the prism axis, while the *X*-axis points toward one of the prism’s edges. The *Z*-axis is perpendicular to both the *X* and *Y* axes, completing the right-handed coordinate system. Six triaxial accelerometers are installed in an alternating pattern on the sides of the prism, with each sensor’s center positioned at the midpoint of the respective hexagonal edge. Specifically, the *y*-axis of each sensor aligns with the global *Y*-axis, while the *z*-axis is perpendicular to the prism’s side, pointing outward. 

In the figure, the hexagonal prism has a height of 2 h and a side length denoted as l. The position coordinates (*L*_x_, *L*_y_, *L*_z_) and alignment angles (*θ*_x_, *θ*_y_, *θ*_z_) of the six sensors’ sensitive axes in the XYZ coordinate system are provided below:

(1) $$\left[\begin{array}{c} L_{x}\\ L_{y}\\ L_{z} \end{array}\right]=\left[\begin{array}{cccccc} 0 & \sqrt{3}l/2 & \sqrt{3}l/2 & 0 & \sqrt{3}l/2 & \sqrt{3}l/2\\ l & l/2 & -l/2 & -l & -l/2 & -l/2\\ h & -h & h & -h & h & -h \end{array}\right]$$

(2) $$\left[\begin{array}{c} \theta_{x}\\ \theta_{y}\\ \theta_{z} \end{array}\right]=\pi \left[\begin{array}{cccccc} 1/2 & 1/2 & 1/2 & 1/2 & 1/2 & 1/2\\ 1/2 & 1/2 & 1/2 & 1/2 & 1/2 & 1/2\\ 1/2 & 1/6 & -1/6 & -1/2 & -5/6 & 5/6 \end{array}\right]$$

### 2.2. Inclined Six-Position Installation Error Calibration Scheme

In traditional accelerometer calibration, the following error model is usually used for calibration:

(3) $$W=\left[\begin{array}{ccc} S_{x}^{a} & 0 & 0\\ 0 & S_{y}^{a} & 0\\ 0 & 0 & S_{z}^{a} \end{array}\right]\left[\begin{array}{ccc} 1 & \alpha_{\mathrm{yx}} & \alpha_{\mathrm{zx}}\\ \alpha_{\mathrm{xy}} & 1 & \alpha_{\mathrm{zy}}\\ \alpha_{\mathrm{xz}} & \alpha_{\mathrm{yz}} & 1 \end{array}\right]G+\left[\begin{array}{l} b_{ax}\\ b_{ay}\\ b_{az} \end{array}\right]$$

where *W* represents the measured output of the accelerometer, *G* (*G* = [0, g, 0]^T^) represents the theoretical output of the accelerometer in the absence of installation errors, *α_ij_* (*i*, *j* = *x*, *y*, *z*) represents installation errors, $S_{i}^{a}$(*i* = *x*, *y*, *z*) represents scale factor errors, *b_ai_* (*i* = *x*, *y*, *z*) represents bias errors.

Since the sensors have already been calibrated and compensated at the factory, the bias errors can be neglected in this context. Thus, the equation can be simplified to Equation (4):

(4) $$W=\left[\begin{array}{ccc} S_{x}^{a} & S_{y}^{a}\alpha_{\mathrm{yx}} & S_{z}^{a}\alpha_{\mathrm{zx}}\\ S_{x}^{a}\alpha_{\mathrm{xy}} & S_{y}^{a} & S_{z}^{a}\alpha_{\mathrm{zy}}\\ S_{x}^{a}\alpha_{\mathrm{xz}} & S_{y}^{a}\alpha_{\mathrm{yz}} & S_{z}^{a} \end{array}\right]G$$

This simplification leads to the following:

(5) G=EW

where the compensation matrix $E={\left[\begin{array}{ccc} S_{x}^{a} & S_{y}^{a}\alpha_{\mathrm{yx}} & S_{z}^{a}\alpha_{\mathrm{zx}}\\ S_{x}^{a}\alpha_{\mathrm{xy}} & S_{y}^{a} & S_{z}^{a}\alpha_{\mathrm{zy}}\\ S_{x}^{a}\alpha_{\mathrm{xz}} & S_{y}^{a}\alpha_{\mathrm{yz}} & S_{z}^{a} \end{array}\right]}^{-1}$.

The traditional method for compensating installation errors involves using Equation (4) to map the actual measured values to their theoretical values at specific measurement points. This process enables the determination of the current installation error matrix and, consequently, the derivation of the compensation matrix *E*. As shown in Equation (5), the compensation matrix *E* is then applied to correct the installation errors, bringing the measured values closer to the true values.

The theoretical output of the accelerometer represents the projection of the gravitational acceleration from the geographic coordinate system (north–east–up) to the drill string coordinate system (b-frame), which can be obtained through coordinate transformation. Therefore, based on Equation (5), the compensation matrix E can be reformulated as follows:

(6) $$E=C_{b}^{n}\left[\begin{array}{c} 0\\ 0\\ g \end{array}\right]W^{-1}$$

where $\left[\begin{array}{c} 0\\ 0\\ g \end{array}\right]$ represents the gravitational acceleration in the geographic coordinate system (local horizontal coordinate system), $C_{b}^{n}$ is the attitude transformation matrix from the geographic coordinate system to the body frame.

By taking the derivative of Equation (6), the relative error expression of the compensation matrix *E* can be obtained as follows:

(7) $$dE=C_{b}^{n}\left[\begin{array}{c} 0\\ 0\\ g \end{array}\right]\cdot dW^{-1}$$

From the relative error expression of the compensation matrix *E*, it is clear that the accuracy of *E* depends on the precision of the measurements. Without improving the intrinsic measurement accuracy of the sensors, variations in the attitude matrix $C_{b}^{n}$ can affect the measurement accuracy, thereby impacting the precision of the compensation matrix. Therefore, by adjusting the attitude matrix $C_{b}^{n}$ using conventional calibration methods, the accuracy of the compensation matrix can be improved, and the issues primarily caused by measurement noise can be mitigated.

Assuming the measured values from a triaxial accelerometer are *g_x_*, *g_y_*, and *g_z_*, the attitude matrix $C_{b}^{n}$ projects the gravitational acceleration into the drill string coordinate system (b-frame). The outputs of the accelerometers along the *x*, *y*, and *z* axes of the drill string can then be represented as follows:

(8) $$\left\{\begin{array}{l} g_{x}=g\sin I\cos T+\mu_{x}\\ g_{y}=g\sin I\sin T+\mu_{y}\\ g_{z}=g\cos I+\mu_{z} \end{array}\right.$$

where *I* represents the inclination angle (the angle between the borehole direction line and the gravity line); *T* represents the toolface angle; and *μ_x_*, *μ_y_*, *μ_z_* are the random noises along the *x*, *y*, and *z* axes, respectively.

By differentiating Equation (8), the relative errors in the three axis measurements *r_x_*, *r_y_*, and *r_z_* can be derived as follows:

(9) $$\left\{\begin{array}{l} r_{x}=\cot IdI-\tan TdT+\mu_{x}\\ r_{y}=\cot IdI+\cot TdT+\mu_{y}\\ r_{z}=-\tan IdI+\mu_{z} \end{array}\right.$$

From Equation (9), it is obvious that the output error of the triaxial accelerometer is related to the cutter face angle and inclination. Assuming that the horizontal axis error is equal to the vertical axis error (*r_x_* = *r_z_*) and only considering the influence of the inclination angle on the measurement error (*T* = 0°), the inclination angle *I* can be calculated as 45°. In order to further determine that the rotation angle is 45° instead of the other angles, we set the degree rotation angle as *θ* for analysis.

After the entire carrier rotates *θ*° around the *x*-axis, the output expression of the three-axis accelerometer can be written as follows:

(10) $$\left\{\begin{array}{l} {\;g}_{x}^{\prime }=g\cos\theta_{x}\sin I\cos T+g\sin\theta_{x}\cos I\pm \mu_{x}\\ {\;g}_{y}^{\prime }=g\sin I\sin T\pm \mu_{y}\\ {\;g}_{x}^{\prime }=-g\sin\theta_{z}\sin I\cos T+g\cos\theta_{z}\cos I\pm \mu_{z} \end{array}\right.$$

Multiplying ${\;g}_{x}^{\prime }$ and ${\;g}_{z}^{\prime }$ by sin *θ* and cos *θ* respectively yields the following:

(11) $$\left\{\begin{array}{l} {\;g}_{x}^{\prime }\sin\theta =g\cos\theta \sin\theta \sin I\cos T+g(\sin\theta {)}^{2}\cos I\pm \sin\theta \mu_{x}\\ {\;g}_{z}^{\prime }\cos\theta =-\mathrm{gsin}\theta \cos\theta \sin I\cos T+g(\cos\theta {)}^{2}\cos I\pm \cos\theta \mu_{z} \end{array}\right.$$

By adding ${\;g}_{x}^{\prime }\sin\theta$ and ${\;g}_{z}^{\prime }\cos\theta$, their geometric values are the same as the true measured values under the orthogonal structure considering errors, i.e.,${\;g}_{x}^{\prime }\sin\theta +{\;g}_{z}^{\prime }\cos\theta =g_{z}$. Therefore, the true measured value of the wellbore inclination angle $\hat{I}$ can be obtained as follows:

(12) $$\hat{I}=\arccos\frac{g\cos I\pm \mu_{x}\sin\theta \pm \mu_{z}\cos\theta }{g}$$

Assuming the measurement error of the accelerometer is 5% of the measured value, i.e., the maximum measurement error $\mu_{x}=\mu_{y}=\mu_{z}=0.005g$, then, we obtain the following:

(13) $$\hat{I}=\arccos(\cos I\pm 0.005\sin\theta \pm 0.005\cos\theta )$$

The calculation error of the wellbore inclination angle is as follows:

(14) $$E_{I}=\hat{I}-I=\arccos(\cos I\pm 0.005\sin\theta \pm 0.005\cos\theta )-I$$

Similarly, by subtracting ${\;g\;\prime }_{x}\sin\theta$ from ${\;g\;\prime }_{z}\cos\theta$, it can be inferred that the calculation error of the tool face angle is the following:

(15) $$E_{T}=\hat{T}-T=\arcsin\frac{\sin I\cos T\pm 0.005\sin\theta \pm 0.005\cos\theta }{\sin I}-T$$

**Figure 2:**
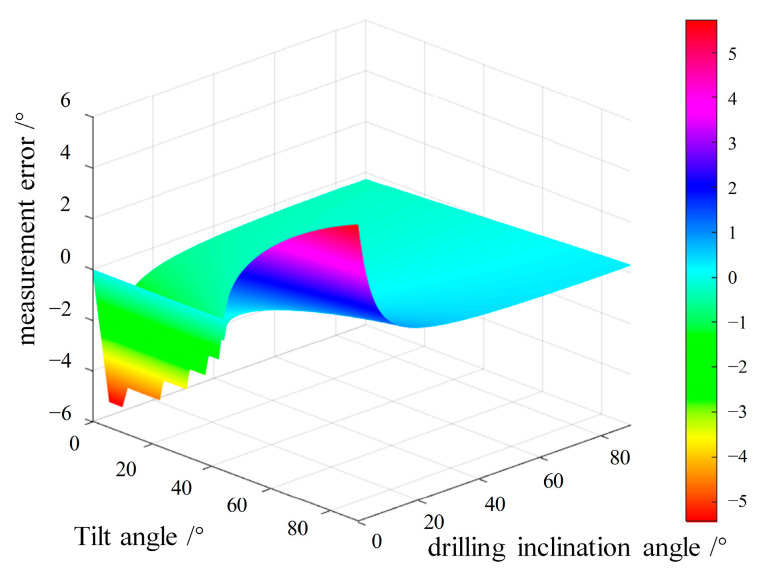
Three-dimensional simulation diagram corresponding to different drilling inclination angles and tilt angles.

By using Formula (14) and setting both the drilling inclination angle and the tilt angle as independent variables, we can draw three-dimensional simulation images corresponding to different drilling inclination and tilt angles, as shown in Figure 2.

From Figure 2, it can be seen that, when the inclination angle is large, the selection of the bias angle is more extensive, while, when the inclination angle is small, only when the tilt angle is 45°, the measurement error is closest to 0.

**Figure 3:**
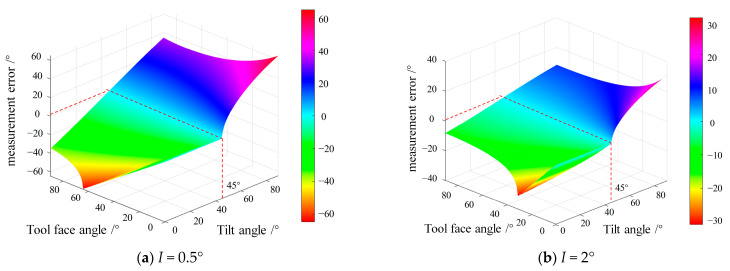
Three-dimensional simulation diagram corresponding to different tool face angles and tilt angles.

From Formula (14), it can be seen that the measurement error of the tool face angle is related to the inclination angle *I*. In order to obtain three-dimensional simulation images corresponding to different tool face angles and offset angles, we set the inclination angles *I* = 0.5° and *I* = 2° as independent variables and then set both the tool face angle and inclination angle as independent variables. The obtained images are shown in Figure 3a,b.

From the figure, it can be seen that, whether at *I* = 0.5° or *I* = 2°, the measurement error of the tool face angle is closest to 0 when the tilt angle is 45°.

In summary, when the tilt angle is 45°, the sensor output state is optimal. Additionally, previous research has shown that accelerometers exhibit better linearity around an inclination angle of 45° [19,20]. So, in order to maximize the sensor output and achieve a more accurate compensation matrix during the calibration process, the new calibration scheme involves rotating the carrier 45° and placing it and selecting specific measurement points under this tilted condition. According to the above research, due to the fact that the coordinate system in this scheme points from the Y-axis to the sky, replacing the position of the Z-axis in the conventional coordinate system, the carrier in this calibration scheme should be rotated 45° around the X-axis. According to the above research, the carrier in this calibration scheme should be rotated 45° around the *X*-axis.

**Figure 4:**
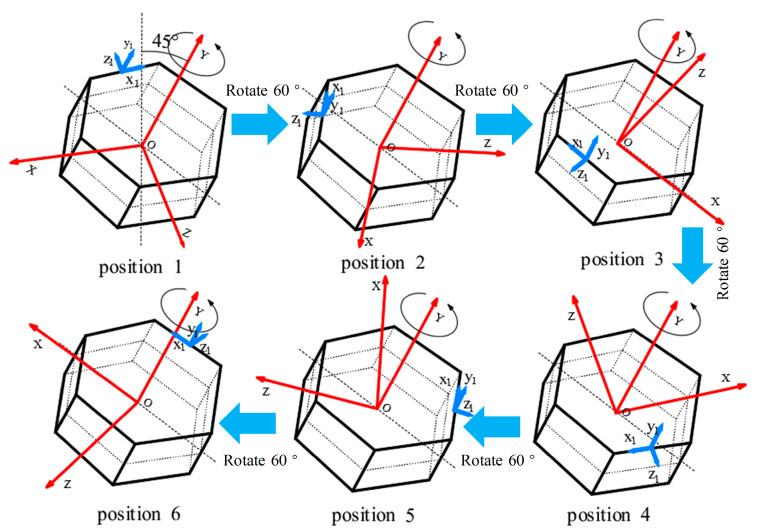
Schematic flow diagram of the six-position calibration method.

Given that our MEMS-IMU redundant configuration follows a hexagonal prism structure, after installing the carrier at a 45° inclination, the carrier rotates around its own axis by 60° each time to determine the measurement point, which ensures that the overall spatial position of the carrier remains consistent before and after each rotation. Consequently, the special measurement points for acquiring actual measurements are sequentially selected at tool face angles of 0°, 60°, 120°, 180°, 240°, and 300°. The schematic flow diagram of this calibration method is illustrated in Figure 4.

From the figure, it can be seen that, from position 1 to position 6, the carrier maintains a 45° tilt around the *x*-axis, and each position changes to the next position by rotating 60° around the *y*-axis.

Given that the structure of the hexagonal prism configuration has been established, the theoretical values can be accurately determined at these six positions using the relative positions of the six sensors, thereby enhancing the accuracy of the calibration. Through theoretical derivation, the theoretical outputs *G* and the measured outputs *W* of the accelerometers for the six MEMS-IMU sensors under the inclined six-position installation error correction scheme can be expressed as follows:

(16) $$G=\left[\begin{array}{cccccc} g_{x1} & g_{x2} & g_{x3} & g_{x4} & g_{x5} & g_{x6}\\ g_{y1} & g_{y2} & g_{y3} & g_{y4} & g_{y5} & g_{y6}\\ g_{z1} & g_{z2} & g_{z3} & g_{z4} & g_{z5} & g_{z6} \end{array}\right]$$

(17) $$G=g\left[\begin{array}{cccccc} 0 & \sqrt{6}/4 & \sqrt{6}/4 & 0 & -\sqrt{6}/4 & -\sqrt{6}/4\\ \sqrt{2}/2 & \sqrt{2}/2 & \sqrt{2}/2 & \sqrt{2}/2 & \sqrt{2}/2 & \sqrt{2}/2\\ \sqrt{2}/2 & \sqrt{2}/4 & -\sqrt{2}/4 & -\sqrt{2}/2 & -\sqrt{2}/4 & \sqrt{2}/4 \end{array}\right]$$

(18) $$W=\left[\begin{array}{cccccc} w_{x1} & w_{x2} & w_{x3} & w_{x4} & w_{x5} & w_{x6}\\ w_{y1} & w_{y2} & w_{y3} & w_{y4} & w_{y5} & w_{y6}\\ w_{z1} & w_{z2} & w_{z3} & w_{z4} & w_{z5} & w_{z6} \end{array}\right]$$

(19) $$W=\left[\begin{array}{cccccc} \mu_{x} & g\sin I\sin T+\mu_{x} & g\sin I\sin T+\mu_{x} & \mu_{x} & g\sin I\sin T+\mu_{x} & g\sin I\sin T+\mu_{x}\\ g\cos I+\mu_{y} & g\cos I+\mu_{y} & g\cos I+\mu_{y} & g\cos I+\mu_{y} & g\cos I+\mu_{y} & g\cos I+\mu_{y}\\ g\sin I\cos T+\mu_{z} & g\sin I\cos T+\mu_{z} & g\sin I\cos T+\mu_{z} & g\sin I\cos T+\mu_{z} & g\sin I\cos T+\mu_{z} & g\sin I\cos T+\mu_{z} \end{array}\right]$$

According to Equations (17) and (19), and by utilizing Equation (5), the compensation matrix *E* can be ultimately determined using the least-squares method.

## 3. Calibration Method Validation

### 3.1. Simulation Experiment

**Figure 5:**
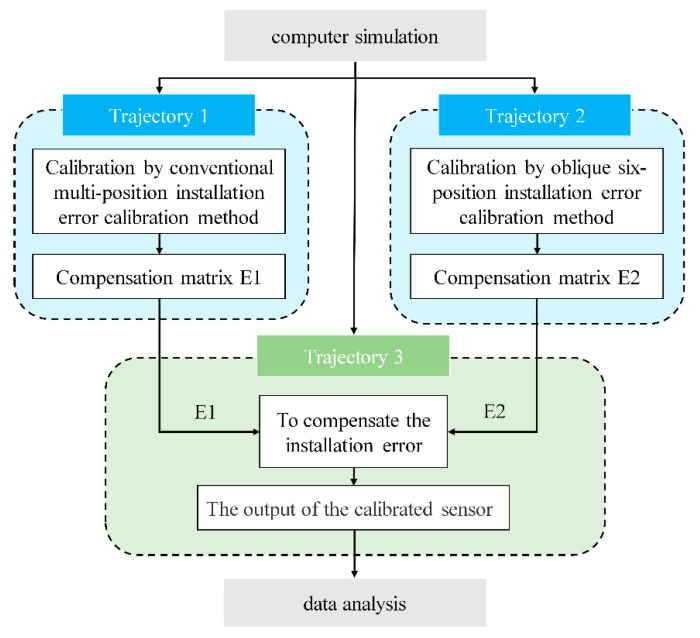
Flowchart of the trajectory simulation.

In this study, simulations were conducted using MATLAB R2022a to compare the performance of the conventional compensation method and the inclined six-position method. The compensation matrices *E*_1_ and *E*_2_ were derived for each respective method. Subsequently, a new motion trajectory was generated, and both the compensation matrices *E*_1_ and *E*_2_ were applied to it. Finally, the accuracy of the two methods was compared by analyzing the sensor outputs after applying the compensation matrices. The entire process is illustrated in Figure 5.

The specific steps of this simulation are as follows:

(1) Simulate the generation of MEMS-IMU Trajectory 1 by sequentially orienting the *x*-axis, *y*-axis, and *z*-axis vertically to obtain three sets of sensor measurements. These measurements serve as theoretical values without installation errors. Subsequently, installation errors (with an error angle of 0.00278° between each pair of axes) were introduced into this trajectory. New measurements were then taken to obtain three sets of values with installation errors, representing the actual values. Using these theoretical and actual values, the compensation matrix *E*_1_ was calculated using the conventional compensation method.
(2) Simulate the generation of a new MEMS-IMU Trajectory 2 in which the sensor moves from its initial state to a position with a 45° inclination angle and a 0° tool face angle, designated as Position 1. The sensor was then moved to five additional positions with tool face angles of 60°, 120°, 180°, 240°, and 300°. The six sets of measurement data acquired from these positions were used as the theoretical values for the inclined six-position installation error method. Subsequently, the same installation errors as in Trajectory 1 were introduced, and new measurements were taken to obtain six sets of values with installation errors, representing the actual values. Using these theoretical and actual values, the six-position compensation matrix *E*_2_ was derived through the least-squares method.
(3) Simulate the generation of a test comparison MEMS-IMU Trajectory 3. The measurements obtained without installation errors were used as the theoretical reference values. Subsequently, the same installation errors were introduced into Trajectory 3. The compensation matrices *E*_1_ and *E*_2_ were then applied separately to correct these errors, and the accuracy of each method was compared based on the compensated outputs.

The sensor errors added when generating IMU trajectories were as follows: the zero bias instability of the accelerometer was 10 μg; the bias stability in the full-temperature domain of the accelerometer was 4 mg; the resolution of the accelerometer was 0.5 mg; the nonlinear accuracy of the accelerometer was 0.1% FS; the random walk of the accelerometer was 0.05 m/s/√h the bandwidth of the accelerometer was 50 Hz; the gyro bias instability was 4 °/h; the zero point accuracy in the full-temperature domain of the gyro was 0.1 °/s; the resolution of the gyro was 0.01 °/s; the nonlinear accuracy of the gyro was 0.1% FS; the random walk of the gyro was 0.3 °/√h; and the bandwidth of the gyro was 50 Hz.

After completing the simulations and obtaining *E*_1_ and *E*_2_, Equation (4) was used to derive the calibration results for both methods, as shown in Table 1.

From the table above, it is observed that, after calibration using the conventional multi-position error calibration method, the average calibrated installation error was approximately 0.005°, with a deviation from the true value of around 0.0047°. In contrast, the inclined six-position compensation method resulted in an overall deviation of approximately 0.0028° from the true value. This result represents a 40.4% reduction in installation error compared with the conventional method, demonstrating the superior performance of the inclined six-position method. However, due to the rotation about the *X*-axis in the calibration scheme, the installation error between the *x*-axis and *z*-axis is not fully compensated in the inclined six-position method. As a result, the accuracy in this aspect is comparable with that of the conventional compensation method.

**Figure 6:**
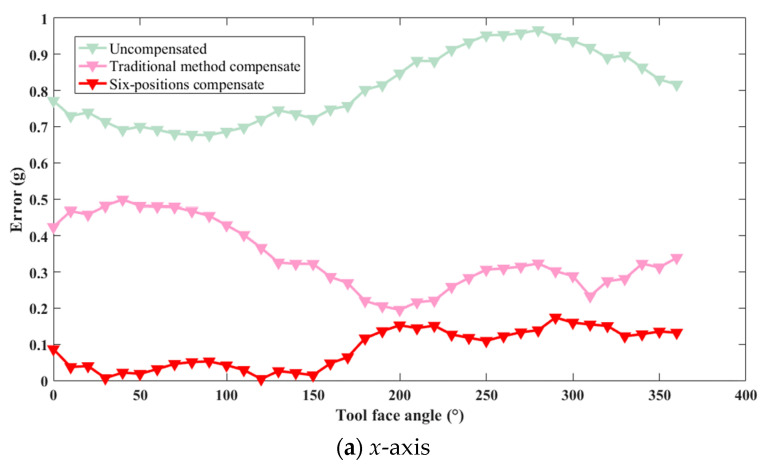

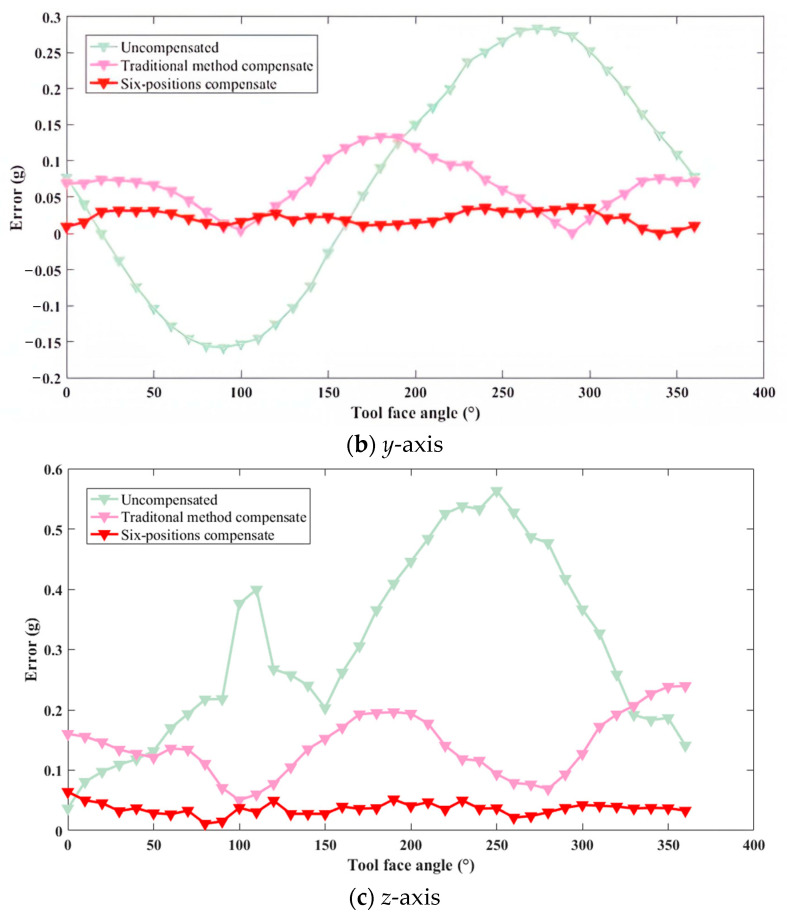
Comparison of accelerometer triaxial accuracy in simulation.

To visually assess the compensation effectiveness, one IMU triaxial accelerometer was selected, and its installation error graphs for the *x*, *y*, and *z* axes were plotted, as shown in Figure 6 below.

As can be seen from the figure, after compensation using both methods, the errors were significantly reduced compared with the original data. Furthermore, the effect of compensation using the oblique six-position compensation method was superior to that of the traditional compensation method. Since the errors added during simulation were the same, and the compensation matrix E primarily compensated for installation errors; it can be concluded that the oblique six-position compensation method was more effective in compensating for installation errors than the traditional method.

### 3.2. Experimental Validation of the Effectiveness of the Inclined Six-Position Calibration Method

**Figure 7:**
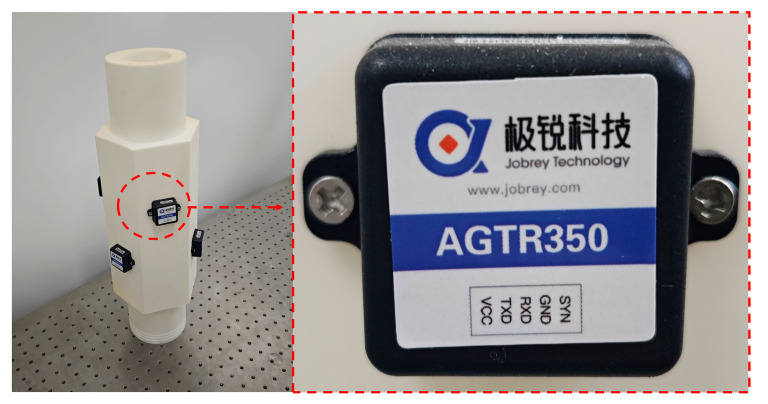
Six-MEMS-IMU redundant system and sensors.

To further validate the effectiveness of this calibration method, a six-MEMS-IMU redundant system with a hollow six prism structure was built by using 3D-printing technology (as shown in Figure 7).

The MEMS sensor selected for this study was the AGTR350 model. It has dimensions of 36 mm × 36 mm × 16 mm, and its specific parameters are detailed in Table 2 and Table 3.

The MEMS sensor array was installed on a triaxial rotary table. Similar to the simulation, the matrices *E*_1_ and *E*_2_ were obtained through a combination of experimental measurements and calculations.

**Figure 8:**
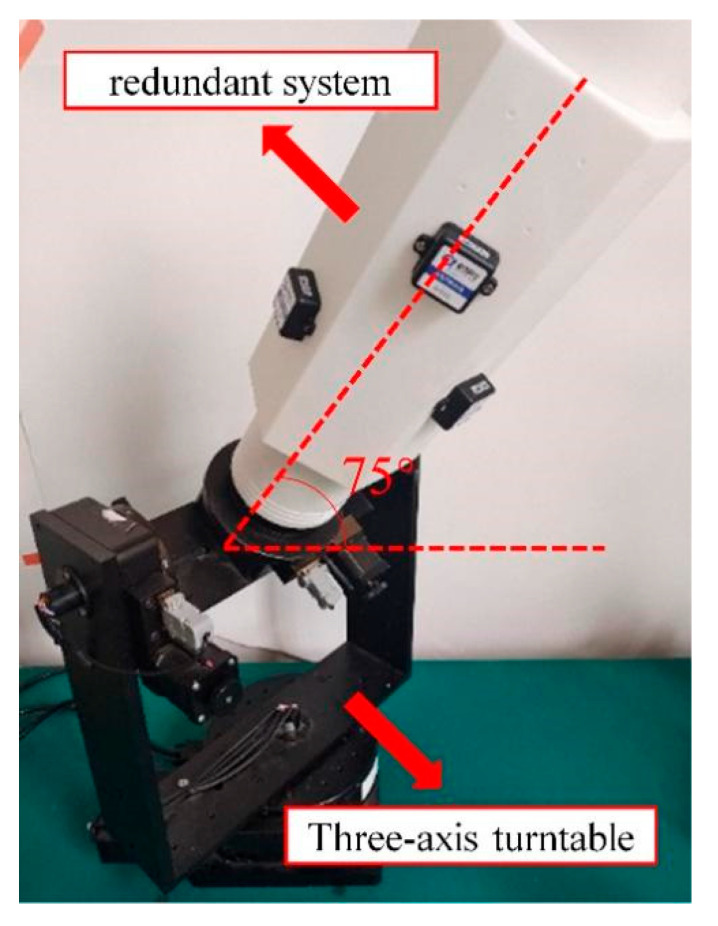
Triaxial rotary table and actual test setup.

**Figure 9:**
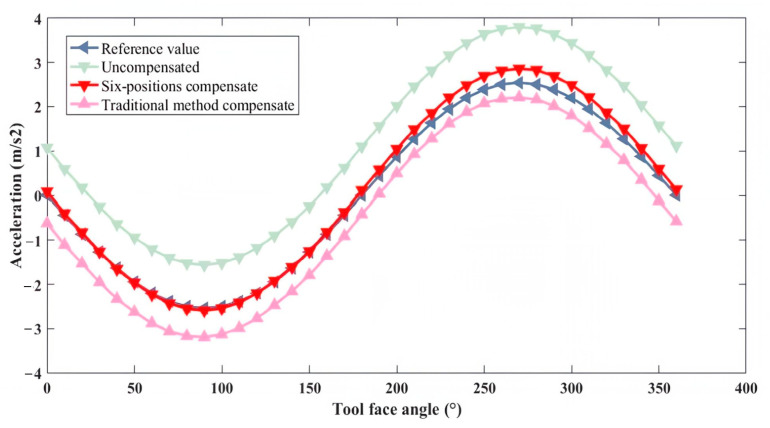
Comparison of the effectiveness of compensation methods in *x*-axis.

**Figure 10:**
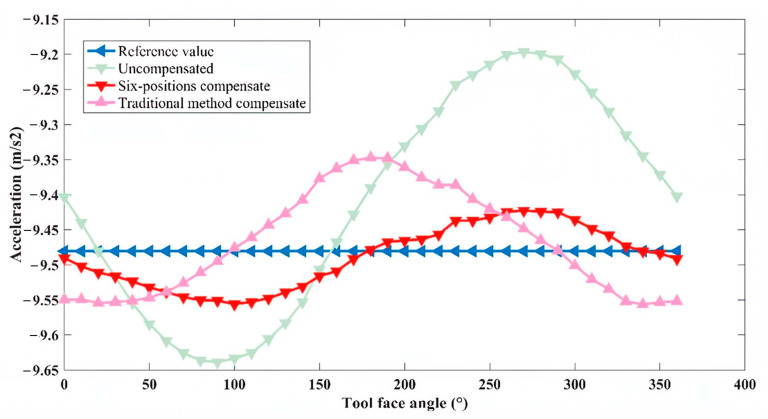
Comparison of the effectiveness of compensation methods in *y*-axis.

**Figure 11:**
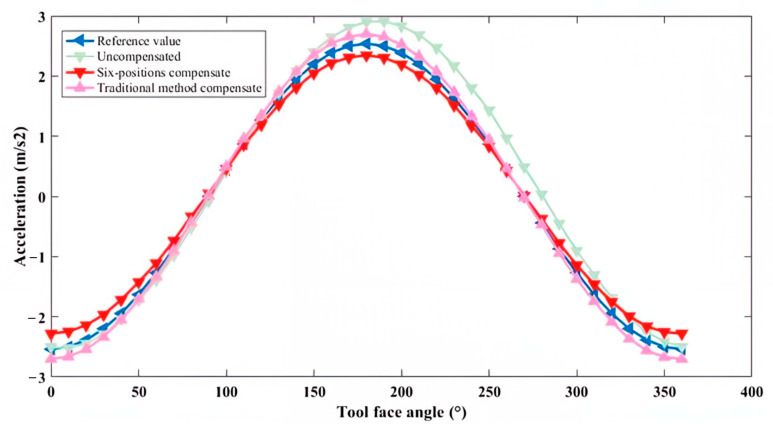
Comparison of the effectiveness of compensation methods in *z*-axis.

After obtaining *E*_1_ and *E*_2_, the triaxial rotary table was adjusted to position the sensor array at a 75° angle with respect to the horizontal plane (as shown in Figure 8). This position was recorded as Measurement Point 1, and the sensor measurements were acquired. Subsequently, the triaxial rotary table was further adjusted to rotate the sensor array around the axis of its hexagonal prism carrier through a full revolution, with measurement points selected every 10°. For one of the six IMUs, the theoretical reference values, uncompensated measurement values, compensated values using the six-position calibration method, and compensated values using traditional calibration methods at these measurement points were compared. The results are illustrated in Figure 9, Figure 10 and Figure 11.

For the *x*-axis, the theoretical values without installation error, the uncompensated measurement values, and the compensated values using both traditional and new methods all exhibited a sinusoidal trend. Over the entire range of tool face angles from 0° to 360°, the new method achieved higher compensation accuracy than the traditional method. For the *y*-axis, assuming the *y*-axis orientation aligned with the drill pipe direction and no installation error was present, the theoretical values were represented by a straight line. Due to the presence of installation errors, the uncompensated measurement values exhibited a sinusoidal trend. After correction using the new method, although the values still exhibited a sinusoidal trend, the amplitude of the error was significantly reduced. On the other hand, after correction using the traditional method, the values followed a cosine trend, indicating lower compensation accuracy than the new method. For the *z*-axis, the uncompensated measurement values, as well as those corrected using both traditional and new methods, closely approximated the theoretical values without installation error. This result suggests that the installation error angle along the *z*-axis direction was negligible.

Using the experimental data, we calculated the peak-to-peak, average, and maximum absolute errors for the measurements along the *x*, *y*, and *z* axes. These results are summarized in Table 4, Table 5 and Table 6.

From the data presented in the previous table, several observations can be made: Firstly, regarding the *Z*-axis, due to the minimal installation error angle of the experimental sensors, the compensation accuracy achieved by both methods was very similar. This indicates that the installation errors along the *Z*-axis were effectively negligible, resulting in minimal discrepancy between the two compensation techniques. Secondly, it can be seen that the variance value of absolute error on the *y*-axis changed significantly. The variance of the absolute error on the *y*-axis after compensation by the new method was minimized, indicating that the sensor output was more convergent. Additionally, for the other two axes (*x* and *y*), the application of traditional compensation methods resulted in an average reduction of the absolute measurement errors by 62% and 54%, respectively. In contrast, employing the inclined six-position compensation method led to a more substantial decrease in the absolute measurement errors, with averages of 83% and 68% reductions for the *x* and *y* axes, respectively. Overall, the utilization of the inclined six-position compensation method demonstrated a significant improvement in sensor accuracy compared with traditional compensation methods, achieving enhancements exceeding 25%. This method thus delivered a notably superior compensation effect.

## 4. Discussion

This study addresses the issue of low accuracy in compensation matrices during the installation error compensation process for redundant inertial navigation systems. To address this, we propose a six-position compensation method based on an inclined installation configuration of six MEMS-IMUs arranged on a hexagonal prism structure. This method effectively mitigates the issue of excessive noise in horizontal axis measurements under conventional methods, achieving significantly improved compensation accuracy and enhancing sensor measurement precision. However, several limitations were identified in this study:

(1) During the correction of installation errors, this study did not account for the influence of temperature. The measurement accuracy of MEMS sensors is significantly affected by temperature variations, with notable performance degradation occurring at elevated temperatures.
(2) When comparing the accuracy of the two compensation methods, the simulated drilling trajectories used were relatively simple. As a result, the compensation accuracy under more complex drilling trajectories remains unverified.
(3) Both the theoretical analysis and experimental validation conducted in this study were performed under static conditions. The compensation accuracy under dynamic conditions has not been investigated.

Future research will focus on addressing these limitations by incorporating additional practical considerations such as temperature effects, validating the method under more complex drilling trajectories, and evaluating its performance under dynamic conditions. These efforts will refine and optimize the proposed method, ensuring its robustness and reliability in real-world applications.

## 5. Conclusions

This study investigates the calibration method for installation errors in redundant inertial navigation systems based on a hexagonal prism structure with six MEMS-IMUs. The following conclusions are drawn:

(1) This study introduces an innovative six-position compensation method that utilizes an inclined installation configuration of six MEMS-IMUs on a hexagonal prism structure. In this new compensation scheme, the carrier is tilted 45° around the *X*-axis. Six specific measurement points are then selected at tool face angles of 0°, 60°, 120°, 180°, 240°, and 300° in this tilted state to derive the compensation matrix. This method effectively addresses the issue of low accuracy in compensation matrices during the installation error compensation process for redundant inertial navigation systems by improving the measurement precision and reducing the impact of noise.
(2) The effectiveness of the proposed method was validated through both simulation and experimental testing. The simulation results demonstrated that after calibration using the inclined six-position compensation method, installation errors were reduced by 40.4% compared with conventional multi-position error calibration methods, highlighting the superior performance of the proposed method. These findings were further supported by practical experiments, in which the absolute measurement errors along the *x* and *y* axes were reduced by 83% and 68%, respectively. Compared with traditional compensation methods, the sensor accuracy improved by more than 25%, demonstrating the superior compensation effect of the proposed method.

## Figures and Tables

**Table 1:** Calibration results of two methods.

Parameter	Conventional Compensation Method	Error from True Value	Inclined Six-Position Method	Error from True Value
α_yx_/rad	0.00250	0.00028	−0.00003	0.00281
α_zx_/rad	−0.04997	0.05275	−0.05108	0.05386
α_xy_/rad	0.05010	0.04732	0.00000	0.00278
α_zy_/rad	0.05000	0.04722	0.00000	0.00278
α_xz_/rad	0.05000	0.04722	0.05138	0.04860
α_xy_/rad	−0.04992	0.05270	0.00059	0.00219

**Table 2:** IMU accelerometer relevant parameters.

Accelerometer	Minimum Value	Typical Values	Maximum Value
Range (g)	−6	-	+6
Zero Bias Instability (μg)	5	10	15
Full-Temperature Bias Stability (mg)	3	4	5
Resolution (mg)	-	0.5	-
Nonlinear Accuracy (%FS)	-	0.1	-
Random Walk (m/s/√h)	0.03	0.05	0.1
Bandwidth (Hz)	-	50	-

**Table 3:** IMU gyroscope relevant parameters.

Gyroscope	Minimum Value	Typical Values	Maximum Value
Range (°/s)	−400	-	+400
Zero Bias Instability (°/h)	3	4	5
Full-Temperature Bias Stability (°/s)	0.08	0.1	0.2
Resolution ((°/s)	-	0.01	-
Nonlinear Accuracy (%FS)	-	0.1	-
Random Walk (°/h)	0.2	0.3	0.5
Bandwidth (Hz)	-	50	-

**Table 4:** Comparison of *x*-axis measurement errors.

Absolute Error of *x*-Axis (m/s)	Peak-to-Peak	Average Value	Maximum Value	Variance
Raw Measurement Data	1.6340	0.8076	0.9579	0.0983
Data Compensated by Traditional Methods	0.6079	0.3053	0.4982	0.1256
Data Compensated by New Methods	0.3157	0.1380	0.3150	0.1318

**Table 5:** Comparison of *y*-axis measurement errors.

Absolute Error of *x*-Axis (m/s)	Peak-to-Peak	Average Value	Maximum Value	Variance
Raw Measurement Data	0.4426	0.1456	0.2841	0.0822
Data Compensated by Traditional Methods	0.2095	0.0656	0.1333	0.0354
Data Compensated by New Methods	0.1584	0.0467	0.1006	0.0224

**Table 6:** Comparison of *z*-axis measurement errors.

Absolute Error of *z*-axis (m/s)	Peak-to-Peak	Average Value	Maximum Value	Variance
Raw Measurement Data	0.3142	0.2448	0.3057	0.1939
Data Compensated by Traditional Methods	0.2031	0.1532	0.2389	0.0795
Data Compensated by New Methods	0.2153	0.1492	0.2069	0.0754

## Data Availability

The original contributions presented in this study are included in the article. Further inquiries can be directed to the corresponding author.
